# Expression Profiles of Chitin Deacetylase Genes and Functional Characterization of Key Genes in *Spodoptera frugiperda*

**DOI:** 10.3390/insects17100997

**Published:** 2026-09-26

**Authors:** Lu Sun, Ying Hu, Minghui Guan, Tonghan Wang, Chengzhi Ye, Yaohui Zheng, Junli Du, Degong Wu

**Affiliations:** 1 College of Resources and Environment, Anhui Science and Technology University, Chuzhou 233100, China; sunluslu@163.com (L.S.); 18756380996@163.com (Y.H.); mhbelucky@163.com (M.G.); ycz2627170492@163.com (C.Y.); 2 College of Agriculture, Anhui Science and Technology University, Chuzhou 233100, China; 18767401521@163.com (T.W.); 19338422575@163.com (Y.Z.); adu83419@163.com (J.D.)

**Keywords:** chitin deacetylase (CDA), *Spodoptera frugiperda*, nanoparticle delivery, RNA interference

## Abstract

Chitin deacetylase (CDA) are critical enzyme involved in the growth and molting of insects. Targeting CDA offers a novel approach for developing environmentally friendly, control strategies against the fall armyworm, *Spodoptera frugiperda* (*S. frugiperda*). In this study, we performed functional characterization of *CDA* genes in this agriculturally important pest. A total of seven *CDA* genes, designated *SfCDA1–7*, were identified. Quantitative real-time PCR (qRT-PCR) was used to systematically profile the expression patterns of these *SfCDA* genes across different developmental stages and tissues. The results showed relatively high transcript levels during the third and fifth instar larval stages, with predominant localization in the integument and midgut. Based on these findings, double-stranded RNA (dsRNA) targeting each *SfCDA* gene was designed, synthesized, and injected into larvae to achieve RNA interference-mediated gene silencing. Knockdown of each *SfCDA* gene via injection revealed that silencing *SfCDA2*, *SfCDA3*, and *SfCDA6* resulted in mortality rates of 16%, 13% and 60%, respectively, whereas knockdown of the remaining genes induced no significant larval mortality. *SfCDA6* exhibited the most pronounced effect. Subsequently, nanoparticle-mediated feeding was employed to silence *SfCDA6*, which led to severe molting defects during the late sixth-instar larva-to-pupa transition, characterized by body shrinkage, developmental arrest, and eventual death, with a mortality rate of 53%. Therefore, *SfCDA6* represents a highly promising molecular target for controlling *S. frugiperda* via RNAi-mediated knockdown. This study clarifies the biological functions of chitin deacetylase genes in *S. frugiperda* molting and development, and identifies *SfCDA6* as a novel potential target for RNAi-based pest management.

## Simple Summary

The fall armyworm is a highly harmful major agricultural pest that seriously threatens the safe production of maize. Long-term reliance on chemical pesticide control not only tends to induce pesticide resistance in pests but also damages the ecological environment. Therefore, there is an urgent need to develop green and efficient alternative prevention and control technologies. Chitin deacetylase genes are crucial for insect molting, growth and development. In this study, we systematically analyzed the activity levels of *SfCDA* genes at different growth stages and in different body parts (midgut, head, integument, fat body, hemolymph, and Malpighian tubules) of the pest, and verified their biological functions through gene-silencing technology. The results showed that *SfCDA6* is significantly involved in the regulation of larval molting. Silencing this gene can severely hinder the processes of larval molting, pupation and adult emergence, cause molting deformities and developmental arrest, and ultimately lead to pest mortality exceeding 53%. This study clarifies the important prevention-and-control potential of this gene, provides high-quality candidate targets for the green prevention and control of the fall armyworm, and has important reference value for developing new eco-friendly pest control technologies and reducing the application of chemical pesticides.

## 1. Introduction

*S. frugiperda*, commonly known as the fall armyworm, belongs to the family Noctuidae within the order Lepidoptera. As a migratory pest, it causes severe agricultural damage worldwide [1]. The primary damage stems from the burrowing behavior and intense feeding activity of the larvae. During the seedling stage, larvae consume leaves and whorls, producing windowpane-like and hole-like lesions. At the ear stage, they bore into tassels and ears, thereby disrupting pollination and grain filling [2]. In severe infestations, such attacks can lead to substantial maize yield losses or even complete crop failure. Characterized by high reproductive capacity, strong resistance to insecticides, and difficulty of control, this pest poses a serious threat to global and domestic food security, causing annual economic losses estimated at 37.6–328.3 billion yuan [3]. Currently, chemical pesticides remain the primary measure for managing this pest. However, long-term, large-scale reliance on a single chemical approach not only accelerates the development of resistance [4] but also endangers ecological balance and the safety of non-target organisms. Driven by China’s policy of reducing pesticide use while improving efficiency, researchers are seeking novel, environmentally friendly control strategies against *S. frugiperda*, particularly RNA interference (RNAi) technology, which has laid a solid foundation for the management of agricultural pests [5].

RNA interference (RNAi) is a process in which endogenous or exogenous double-stranded RNA (dsRNA) induces the silencing of target mRNA sequences [6]. This results in loss of function of the targeted gene, which in turn affects normal insect growth and development; in severe cases, it leads to death [7]. In recent years, RNAi has been widely applied in functional genomic studies of insect pests and has made important strides in validating target gene functions. For example, Arakane and colleagues performed RNAi on nine chitin deacetylase genes in *Tribolium castaneum*. They found that injection of dsRNA targeting *TcCDA1* or *TcCDA2* interfered with molting and eclosion, resulting in incomplete shedding of the old cuticle, adult malformation, and significantly elevated mortality [8]. Zhu et al. injected dsRNA targeting *PxCht5* and *PxCht10* into third-instar larvae of *Plutella xylostella*. Both treatments induced high mortality but produced distinct lethal phenotypes. Larvae subjected to *PxCht5* silencing failed to enter the molting stage and died prior to molting, whereas those with *PxCht10* knockdown reached the molting stage but, after head capsule splitting, could not completely shed the old cuticle and eventually died [9]. Guan and colleagues employed plant-mediated RNAi targeting the *MsCht* gene in *Melanaphis sacchari*. This approach caused molting failure, underdeveloped legs, and abnormal cuticle formation. Affected individuals died due to loss of mobility and energy depletion, while the few survivors exhibited developmental malformations, such as missing leg structures or abnormal cuticle coloration [10]. The order Coleoptera provides well-documented examples. In *Monolepta hieroglyphica*, silencing *MhVATPA* and *MhCOPIβ’* increased mortality by 60–80% [11]. For *Leptinotarsa decemlineata* larvae, injection of dsRNA targeting *SERCA* caused 100% mortality, whereas dsRNA targeting *CPR* reduced body weight by 59% [12]. Collectively, these studies demonstrate that selecting critical genes involved in pest growth and development as targets, and silencing them via RNAi, can disrupt physiological functions and achieve pest control.

Chitin occurs in insects, fungi, and crustaceans, but is absent in vertebrates. In insects, it is a major component of the cuticle, tracheal system, and peritrophic matrix, maintaining structural integrity and physiological function. Chitin metabolism is essential for growth, development, and molting [13]. Chitin deacetylases (CDAs) belong to the carbohydrate esterase 4 (CE4) family [14] and are key enzymes involved in chitin modification. They catalyze the deacetylation of chitin to form chitosan, thereby participating in the structural modification and assembly of the body wall and peritrophic matrix [15]. Studies have shown that aberrant expression of *CDA* genes disrupts normal molting and impairs growth and development [16]. *CDA* was first identified in the midgut of *Trichoplusia ni* in 2005, where its expression was detected only during the larval feeding period and ceased when feeding stopped [17]. Using quantitative real-time PCR (qRT-PCR), Quan et al. reported that *CfCDA2a* and *CfCDA2b* transcripts were significantly upregulated during the molting stage [18]. Xi and colleagues experimentally confirmed that *NlCDA1*, *NlCDA2*, and *NlCDA4* exhibited high expression levels in the cuticle [19]. Sun observed that *BmCDA1* was highly expressed in the cuticle of *Bombyx mori*, with lower expression in the fat body and Malpighian tubules [20]. Yan found that in *Hyphantria cunea*, *HcCDA2* showed expression in the head and integument, reaching a maximum on days 1 and 2 of the fifth instar [21]. Therefore, targeting CDAs to regulate insect chitin modification represents a scientifically sound strategy for pest control. RNAi-based functional validation experiments have further confirmed the crucial role of CDAs in insect molting. In *Oxya chinensis*, injection of dsRNA targeting *OcCDA2* and *OcCDA2A* led to significant downregulation of the target genes, accompanied by delayed molting, morphological deformities, molting failure, and eventual death [22]. Silencing *LdCDA2* in *Leptinotarsa decemlineata* caused 60% pupal mortality [23]. Wu demonstrated in *Tenebrio molitor* that knockdown of either *TmCDA1* or *TmCDA2* was lethal during molting, indicating that both genes regulate pupal molting in this species [24]. In insects such as *Oxya chinensis*, *Tribolium castaneum* [8], and *Bombyx mori* [20], injection-based RNAi has been successfully applied for functional characterization of *CDA* genes. However, naked dsRNA is prone to degradation by nucleases in the environment and is poorly absorbed by the insect midgut, which limits the efficiency of RNAi applications [25]. Chitosan nanoparticles (CNPs) have been shown to effectively protect dsRNA from degradation [26]. At present, a systematic analysis of chitin deacetylase genes in *S. frugiperda* is lacking. In this study, we focused on the *SfCDA* genes. Injection-based RNAi was first used to screen for highly effective interference targets. Subsequently, chitosan nanoparticle-mediated feeding RNAi was employed to elucidate the biological functions of *SfCDA* genes during larval–pupal metamorphosis. Our findings provide a theoretical basis for RNAi research targeting *CDA* genes and fundamental data for developing environmentally friendly control strategies against *S. frugiperda*.

## 2. Materials and Methods

### 2.1. Insect and Plant Materials

The insect specimens used in this study were continuously reared in the maize research laboratory of Anhui Science and Technology University. Both larval and adult stages were maintained in an artificial climate chamber under controlled conditions: temperature at 25 ± 1 °C, relative humidity between 60% and 70%, and a photoperiod of 16 h light:8 h dark (16L:8D). Adults were supplied with a 10% honey solution and provided with fresh maize leaves for oviposition. Throughout the entire rearing period, no chemical insecticides were applied to the experimental insects. The maize line used was the inbred line AK36, and individuals with uniform growth were selected for subsequent experiments.

### 2.2. Identification and Analysis of SfCDA Genes

To identify members of the CDA family in *S. frugiperda*, the genome assembly file (GCA_023101765.3) of this species was retrieved from the NCBI database (https://www.ncbi.nlm.nih.gov, accessed on 10 March 2025). A BLAST search was performed using TBtools software (v2.042) [27] with an E-value threshold set to 1 × 10^−5^. Subsequent domain validation was conducted to ensure the reliability of the obtained results [28]. Physicochemical properties were analyzed using ExPASy (https://web.expasy.org/protparam/, accessed on 13 March 2025) [29]. Subcellular localization was predicted using the WoLF PSORT website (https://wolfpsort.hgc.jp/, accessed on 13 March 2025) [30]. Based on the chromosomal positions of the identified genes, a distribution map along the chromosomes was constructed.

### 2.3. Gene Structure, Conserved Motif Prediction, and Structural Analysis of SfCDA Genes

Gene structure visualization of *SfCDA* genes was performed using TBtools (v2.042). Conserved motif analysis of the SfCDA proteins was carried out via the MEME (v5.5.5) online suite (https://meme-suite.org/meme/, accessed on 20 March 2025), with the number of motifs preset to 10 and all other parameters kept at their default values [31]. Conserved protein domains were identified using the NCBI CDD and SMART (v9.0) databases.

### 2.4. Phylogenetic Tree Construction and Interspecies Synteny Analysis of SfCDA Genes

The sequences of *Drosophila melanogaster*, *Spodoptera litura*, *Helicoverpa armigera*, and *Bombyx mori* were downloaded from public databases using their accession numbers obtained from previous studies [32,33,34,35]. Multiple sequence alignment of protein sequences was carried out in MAFFT version 7.505, and Guidance2 was adopted to evaluate the reliability of the aligned dataset. A maximum-likelihood phylogenetic tree was constructed in MEGA 11.0.13 with 1000 bootstrap replicates [36] and visualized using the iTOL online platform (https://itol.embl.de/, accessed on 6 June 2025) [37]. Synteny analysis was performed using the One Step MCScanX function within TBtools to generate synteny files [27], followed by visualization with the Multiple Synteny Plot tool of TBtools. The resulting figures were refined using Adobe Illustrator 2025.

### 2.5. Transcriptomic Expression Analysis of SfCDA Genes

Transcriptomic data were retrieved from the NCBI SRA database under the BioProject accession numbers PRJNA590312, PRJNA107035 and PRJNA940659. These datasets cover different developmental stages, various tissues and insecticide treatments. Raw RNA-seq reads from these three independent BioProjects were downloaded. FastQC (v0.12.1) was used to assess read quality, and Trimmomatic (v0.40) was applied for adaptor trimming and quality filtering with the parameters SLIDINGWINDOW:4:15 and MINLEN:36. Clean reads were mapped to the *S. frugiperda* reference genome using HISAT2 (v2.2.1). Transcript quantification was performed with StringTie (v2.2.1), and FPKM values were calculated to estimate gene expression levels [38]. Heatmap visualization of gene expression profiles was conducted using the Heatmap plugin embedded in TBtools [39].

### 2.6. Total RNA Extraction, cDNA Synthesis and qRT-PCR

Specimens of *S. frugiperda* were collected at different developmental stages: eggs (*n* = 80), first-instar larvae (*n* = 20), second-instar larvae (*n* = 15), third-instar larvae (*n* = 10), fourth-instar larvae (*n* = 9), fifth-instar larvae (*n* = 9), sixth-instar larvae (*n* = 9), pupae (*n* = 3), and adults (*n* = 3). All larval samples were strictly collected on the first day after molting into the corresponding instar to minimize transcriptional variation within each instar. For tissue sampling, fifth-instar larvae were dissected to isolate the midgut, head, integument, fat body, hemolymph, and Malpighian tubules. Each sample was prepared in three biological replicates. All specimens were rapidly frozen in liquid nitrogen and subsequently stored at −80 °C. Total RNA was extracted from each sample using the TRIzol reagent. Reverse transcription to synthesize cDNA was performed using the HiScript III RT SuperMix for qPCR kit (Vazyme, Nanjing China), and the resulting products were stored at −20 °C until use.

qRT-PCR was used to detect the expression profiles of *SfCDA* genes in different samples, and *SfRPL3* was selected as the reference gene for normalization of target gene expression [40]. The amplification was performed using ChamQ Universal SYBR qPCR Master Mix (Vazyme Biotech, Nanjing China) on a ViiA™ 7 Real-Time PCR System (Applied Biosystems, Foster City, CA, USA). Primer specificity was verified by both melting-curve analysis and agarose gel electrophoresis to ensure single specific amplicons. The qRT-PCR reaction was prepared in a total volume of 10 µL, containing 5 µL of 2× SYBR qPCR Master Mix, 0.2 µL of forward primer, 0.2 µL of reverse primer, 2 µL of cDNA template, and 2.6 µL of ddH_2_O. The thermal cycling program was as follows: initial denaturation at 95 °C for 30 s, followed by 40 cycles of 95 °C for 10 s and 60 °C for 30 s. The amplification efficiency of each primer pair was evaluated, and all values fell within the acceptable range of 90–110%. Each sample included three biological replicates with three technical replicates per biological replicate. The relative expression levels of target genes were calculated using the 2^−ΔΔCt^ method [41] (Table 1).

### 2.7. RNAi of SfCDA Genes

#### 2.7.1. In Vitro Synthesis of dsRNA

Specific primers targeting the *SfCDA* and *GFP* genes were used to amplify the dsDNA templates (Table 2). The ds*GFP* fragment was amplified with T7 promoter-tagged primers designed as previously reported and used as the negative control in RNA interference assays [42]. The resulting PCR products were purified using the FastPure Gel DNA Extraction Mini Kit (Vazyme, Nanjing, China). In vitro transcription was performed using the T7 High Yield RNA Synthesis Kit (Yugong Biotech, Lianyungang, China) with incubation at 37 °C for 6 h. The transcribed products were then digested with DNase I and precipitated with sodium acetate. After purification, the dsRNA was dissolved in RNase-free H_2_O, quantified, and stored at −80 °C.

#### 2.7.2. Preparation of Chitosan Nanoparticles

Chitosan nanoparticles (CNPs) were synthesized via an ionic crosslinking method [43]. Chitosan (degree of deacetylation ≥ 85%) was dissolved in 1% glacial acetic acid. Sodium tripolyphosphate (purity 98%) was prepared as 1.0% *w*/*v* aqueous solution, which was slowly added drop-wise under magnetic stirring at room temperature to form a nanoparticle suspension. The resulting nanoparticles were collected by centrifugation at 8000 rpm for 10 min at 4 °C, dried to obtain chitosan nanopowder, and stored at 4 °C until use. Prior to application, the powder was redissolved in 1% glacial acetic acid and autoclaved to produce a chitosan nanoparticle stock solution. The stock solution was mixed with dsRNA, and the optimal mass ratio of CNP:dsRNA was fixed at 3:1. The chitosan nanoparticles used in this study had an average particle size of 15.2 nm, with a zeta potential of 16.7 mV. All complex solutions were strictly prepared according to the fixed ratio to ensure consistent formulation conditions throughout the feeding and injection experiments. The formation of CNP-dsRNA complexes was verified by 1% agarose gel retardation assay.

#### 2.7.3. RNA Interference via Injection

Larvae of uniform developmental stage were selected. dsRNA targeting each *SfCDA* gene and ds*GFP* (control) were diluted to a concentration of 1500 ng/μL and individually mixed with chitosan nanoparticle solution for encapsulation. Chitosan nanoparticles were mixed with dsRNA to slow dsRNA degradation during in vitro sample preparation and incubation. A Burkard PDE0003 manual microapplicator (Burkard, Rickmanswort, UK) was used to inject the larval abdomen. To ensure consistent experimental conditions and eliminate developmental-stage confounding factors, all seven *SfCDA* genes were subjected to RNAi using uniform day-1 fifth-instar larvae. Before injection, the larvae were chilled on ice to reduce mobility, and each then received 2 μL of the CNPs-encapsulated dsRNA. The treatment group received CNPs-ds*SfCDA*, while the control group received CNPs-ds*GFP*. Three biological replicates were performed per treatment, with ten larvae per replicate. Samples were collected at 24 h, 48 h, and 72 h post-injection, and the expression levels of *SfCDA* genes were measured by qRT-PCR. To evaluate whether ds*SfCDA6* knockdown induces cross-silencing within the *SfCDA* gene family, larval samples were collected at 72 h post-injection from the ds*SfCDA6* treatment group and ds*GFP* control group. Three biological replicates were established for each group, with three larvae pooled per replicate. qRT-PCR was performed to quantify the transcript levels of all other *SfCDA* family genes, to assess whether ds*SfCDA6* treatment non-specifically suppresses the expression of other homologous *SfCDA* genes.

#### 2.7.4. RNA Interference of the *SfCDA6* Gene via Feeding

Third-instar larvae were starved for 12 h prior to the feeding assay. Both dsRNA targeting *SfCDA6* and ds*GFP* were diluted to 2500 ng/μL and mixed with an equal volume of CNPs for encapsulation. A 15 μL aliquot of the CNPs-encapsulated dsRNA was evenly spread onto the surface of a fresh maize leaf piece (1 cm × 3 cm). After air drying, the leaf was offered to the larvae. Following 24 h of feeding, the leaf was replaced with a freshly treated one; after another 48 h, the larvae were fed with untreated normal maize leaves. Larval samples from both the treatment and control groups were collected at 4 h, 8 h, 12 h, 24 h, 48 h, 72 h, 96 h, and 120 h after the start of feeding. These samples were then used as templates for qRT-PCR analysis.

### 2.8. Statistical Analysis

Statistical tests were performed using SPSS Statistics 26.0, and data visualization was conducted with GraphPad Prism 9.5.0. Normality and homogeneity of variance were tested for qRT-PCR gene expression datasets. One-way ANOVA followed by Tukey’s multiple-comparison test (*p* < 0.05) was used to analyze the expression differences in *SfCDA* genes across different developmental stages and tissues. For RNA interference efficiency data, two-way ANOVA was applied to evaluate the effects of treatment (ds*SfCDA* and ds*GFP)* and sampling time on gene knockdown levels, with Sidak’s post hoc multiple comparison test (*p* < 0.05). Relative gene expression levels were calculated using the 2^−ΔΔCt^ method [41]. Larval survival rates of *S. frugiperda* were calculated as the percentage of surviving individuals at designated time points after treatment. Kaplan–Meier analysis was performed for survival data, and differences between survival curves were assessed using the log-rank (Mantel–Cox) test. Statistical significance was set at *p* < 0.05.

## 3. Results

### 3.1. Identification and Analysis of the SfCDA Gene Family

A total of seven chitin deacetylase-like genes were identified in *S. frugiperda*, and these genes are distributed across five chromosomes (Figure 1). They were named based on conserved domain architecture and phylogenetic analysis. Two *CDA* genes are located on chromosome 1 and two on chromosome 14, whereas chromosomes 5, 15, and 28 each harbor a single *CDA* gene. No *CDA* genes were found on the remaining chromosomes. Notably, the two genes on chromosome 1 arise from tandem duplication, as supported by gene sequence similarity and MCScanX synteny analysis.

Physicochemical property analysis revealed that the amino acid lengths of these proteins range from 381 to 2469 aa, with most being approximately 500 aa. Their molecular masses span from 62,721.42 to 278,460.2 Da, and the charge states of these proteins vary under different pH conditions. Except for *SfCDA5*, which exhibited basicity, all other CDA proteins have isoelectric points below 7, indicating an acidic nature. The instability index ranged from 36.74 (*SfCDA4*) to 57.78 (*SfCDA5*). Among them, *SfCDA6* and *SfCDA4* were classified as stable proteins (instability index < 40), whereas the others were unstable (instability index > 40). The aliphatic index values were mostly around 65. Analysis of the grand average of hydropathicity (GRAVY) showed negative values for all proteins, indicating that these family members are generally hydrophilic (Table 3).

### 3.2. Gene Structure, Conserved Motif and Conserved Domain Analyses of SfCDA Genes

Examination of gene architecture, conserved motifs, and domains revealed substantial variation in the numbers of exons and introns (Figure 2A). Across the seven genes, exon lengths ranged from 46 to 2810 bp, and intron lengths from 78 to 5327 bp. Analysis using the MEME website indicated that most conserved motifs exhibited only minor length variation. Further investigation of the distribution of these motifs among SfCDA proteins showed that while most CDA proteins contained ten conserved motifs, *SfCDA3* and *SfCDA2* lacked Motif 10, and *SfCDA6* and *SfCDA4* lacked Motif 9 (Figure 2B). These SfCDA proteins exhibited highly conserved core motifs, although several paralogues showed partial motif loss. All family members possess the CE4_CDA_like domain (Figure 2C), and proteins within this gene family shared highly similar domain organization. This domain belongs to the carbohydrate esterase family 4 (CE4), which possesses the catalytic activity responsible for chitin deacetylation.

### 3.3. Phylogenetic and Synteny Analysis of SfCDA Genes

Based on phylogenetic analysis, the *CDA* gene family in *S. frugiperda* was classified into five subclades. Group I and Group II proteins contain a polysaccharide deacetylase-like catalytic domain, a chitin-binding peritrophin-A domain (ChBD), and a low-density lipoprotein receptor class A domain (LDLa). Group III and Group IV possess the ChBD and CDA catalytic domains but lack the LDLa domain. Group V harbors only a single polysaccharide deacetylase-like catalytic domain. Among these subgroups, Group I contains the largest number of *SfCDA* genes (three in total). Within Group I, one pair of orthologs was identified between *S. frugiperda* and *S. litura*, and another pair between *S. frugiperda* and *H. armigera*. In Group II, one SfCDA gene was found to have an ortholog in *S. litura*. No *SfCDA* genes were found in Group III. Group IV contains two *SfCDA* genes, with two pairs of orthologs identified between *S. frugiperda* and *S. litura* in this subgroup. One pair of orthologs exists between *S. frugiperda* and *S. litura* in Group V (Figure 3). Collectively, the *SfCDA* genes show close homology with those of other major noctuid agricultural pests, including *S. litura* and *H. armigera* (Figure 4).

### 3.4. Transcriptomic Analysis of SfCDA Genes

Developmental stage expression profiles (Figure 5A) revealed that *SfCDA3* and *SfCDA2* maintained high transcript levels throughout all developmental stages. *SfCDA6* was markedly upregulated from the third to fifth larval instars. *SfCDA1* and *SfCDA4* were expressed predominantly during the pupal stage and the fourth instar, respectively, whereas *SfCDA5* and *SfCDA7* exhibited relatively low expression across all stages. Distinct developmental expression patterns were observed among different SfCDA family members.

Further tissue-specific expression analysis revealed pronounced tissue preference among family members (Figure 5B). *SfCDA2* and *SfCDA3* were highly expressed in the head and integument. *SfCDA6* was highly expressed in the midgut. *SfCDA4* and *SfCDA5* displayed similar expression patterns, also elevated in the head and integument, while their expression levels were significantly lower in other tissues such as the hemolymph, Malpighian tubules, midgut, and fat body. Notably, *SfCDA1* and *SfCDA7* maintained low basal expression across all examined tissues without exhibiting any clear tissue preference.

Taken together, *SfCDA2*, *SfCDA3*, and *SfCDA6* showed relatively stable expression, whereas *SfCDA1*, *SfCDA4*, *SfCDA5*, and *SfCDA7* displayed comparatively lower transcript levels.

Analysis of transcriptomic data following exposure to four insecticides (tetrazolidamide, emamectin benzoate, spinetoram, and cyantraniliprole) (Figure 5C) indicated generally low expression of SfCDA family members. Among them, *SfCDA6* exhibited relatively stable expression in response to tetrazolidamide, emamectin benzoate, and cyantraniliprole. *SfCDA7* showed increased transcript abundance by emamectin benzoate and spinetoram. *SfCDA5* showed elevated expression only under tetrazolidamide treatment. *SfCDA1*, *SfCDA2*, *SfCDA3*, and *SfCDA4* displayed similar expression patterns following exposure to emamectin benzoate and cyantraniliprole. Collectively, distinct expression patterns of *SfCDA* genes were observed under different insecticide treatments.

### 3.5. qRT-PCR Analysis of SfCDA Genes

#### 3.5.1. Expression of *SfCDA* Genes at Different Developmental Stages

According to the qRT-PCR results, *SfCDA2* and *SfCDA3* shared a similar expression profile, with transcript levels peaking in third-instar larvae, followed by the adult stage. The expression patterns of *SfCDA1* and *SfCDA7* were closely related; both reached their highest levels in the fifth instar and showed lower levels in the sixth instar, while maintaining relatively low expression across other developmental stages. *SfCDA5* exhibited maximal expression in pupae, with moderate levels in adults and third-instar larvae. *SfCDA4* displayed relatively high expression only in third-instar larvae. Notably, *SfCDA6* was sharply upregulated from the third to sixth larval instars, with expression levels substantially exceeding those observed in other stages, and two distinct peaks occurred, particularly in the third and sixth instars. This expression trend was partially consistent with the spatiotemporal transcriptomic data, while some differences were observed between the two datasets, suggesting that this gene may fulfill an essential physiological function during the late larval developmental period (Figure 6).

#### 3.5.2. Tissue Expression of *SfCDA* Genes

Tissue expression analysis revealed highly similar patterns for *SfCDA2*, *SfCDA3*, and *SfCDA4*, all showing elevated transcript levels in the integument. In contrast, *SfCDA1*, *SfCDA5*, and *SfCDA7* maintained low expression across all examined tissues. Notably, *SfCDA6* exhibited high expression in both the midgut and the integument (Figure 7). Overall, four of the seven *SfCDA* genes (*SfCDA2*, *SfCDA3*, *SfCDA6*, and *SfCDA4*) were highly expressed in the integument, with *SfCDA6* additionally showing high abundance in the midgut, while the remaining three genes (*SfCDA1*, *SfCDA5*, and *SfCDA7*) exhibited low expression levels in all tissues examined. This tissue-specific expression pattern supports the notion that members of the SfCDA family play distinct physiological roles in *S. frugiperda*.

### 3.6. Functional Analysis of SfCDA Genes by RNAi

#### 3.6.1. Silencing Efficiency of *SfCDA* Genes via Injection-Based RNAi

Agarose gel electrophoresis was performed to verify the formation of CNP-dsRNA complexes, using naked dsRNA as a control. The naked dsRNA migrated normally in the gel. In contrast, the encapsulated CNPs-dsRNA complex remained within the loading wells due to reduced electrophoretic mobility (Figure 8, lanes 1–3).

After injection of CNPs-dsRNA (1500 ng/µL) targeting individual *SfCDA* genes into *S. frugiperda* larvae, qRT-PCR results (Figure 9) showed that each dsRNA treatment specifically reduced the transcript level of its corresponding target gene compared with the ds*GFP* control group. Among all tested genes, knockdown of *SfCDA6* produced the strongest silencing effect and the most pronounced phenotypic defects. For *SfCDA6*, the silencing efficiency was 78% at 48 h, and expression dropped to its lowest level at 72 h (16% of the control), corresponding to 84% silencing efficiency. At 72 h post-injection, qRT-PCR further confirmed that the transcript levels of other SfCDA family genes were not significantly altered in the ds*SfCDA6* group relative to the ds*GFP* control, indicating no non-specific cross-silencing among paralogous *SfCDA* genes (Figure 10). Knockdown of *SfCDA2* and *SfCDA3* caused low larval mortality, whereas silencing of *SfCDA1*, *SfCDA4*, *SfCDA5*, and *SfCDA7* did not induce obvious growth retardation or increased mortality, with larval development comparable to that of the control group. In summary, injection of ds*SfCDA* effectively silences *CDA* genes in *S. frugiperda* larvae, yet the silencing efficiency and duration vary in a gene-specific manner.

#### 3.6.2. Phenotypic Changes Following Injection of *SfCDA* dsRNA

After silencing *SfCDA* genes via dsRNA injection, the molting, pupation, and adult emergence processes of *S. frugiperda* were markedly disrupted, exhibiting multi-stage developmental abnormalities. 

During the molting process, treated larvae were able to split the head capsule along the ecdysial line and shed the cuticle of the head and part of the thorax. However, when molting progressed to the mid-abdomen and posterior segments, the larvae exhibited continuous, violent body contractions but failed to fully shed the old cuticle. Consequently, the exuvium formed a ring-like constriction between the old and new bodies (Figure 11A,B). The larvae remained in a semi-ecdysed state: the anterior part of the new body was soft and exposed, whereas the posterior part remained tightly encased in the shriveled old cuticle. This incomplete molting ultimately led to larval death. By contrast, the ds*GFP* control larvae completed molting smoothly without any abnormalities.

At the pupation stage, treated larvae at the end of their final instar could not properly shed the old cuticle, resulting in adhesion between the exuvium and the newly formed pupa. The pupal body was unable to fully extricate itself and eventually died (Figure 11D). In those individuals that did manage to cast off the cuticle, the pupae appeared shrunken with associated interstitial fluid leakage and failed to emerge as adults (Figure 11C). Control pupae were elongate-oval, with a hard, smooth surface, slightly tapered at both ends, and were free of any remnants of the old cuticle.

At the adult emergence stage, treated adults were significantly smaller than controls and displayed general developmental deficiencies, including body distortion, abnormally curved abdomens, twisted and shriveled antennae, as well as severely folded and fused wings that could not unfold, resulting in a complete loss of flight capacity (Figure 11E,F). Control adults exhibited normal morphology and robust locomotor activity. Collectively, silencing of *SfCDA* genes effectively disrupted the metamorphic development of *S. frugiperda*, leading to molting failure, blocked pupation, and eclosion malformations, ultimately causing death.

No developmental defects were observed following knockdown of *SfCDA1*, *SfCDA4*, *SfCDA5*, and *SfCDA7*. In contrast, silencing of *SfCDA2*, *SfCDA3*, and *SfCDA6* resulted in molting-related mortality rates of 16%, 13%, and 60%, respectively (Figure 12).

#### 3.6.3. Silencing Efficiency of *SfCDA6* via Feeding RNAi

Based on previous transcriptomic differential analysis, phenotypic observations following dsRNA injection, qRT-PCR validation, and screening for key larval phenotypes after injection, the *SfCDA6* gene was selected for subsequent feeding-based RNA interference experiments. The results showed that feeding with CNPs-ds*SfCDA6* led to sustained and significant suppression of the target gene. Compared with the CNPs-ds*GFP* control group, transcript levels in treated larvae were markedly reduced at 4, 8, 12, 24, 48, and 72 h post-feeding. The silencing effect weakened from 96 h onward, with expression recovering by 120 h, but declined again during the late sixth-instar larval stage (Figure 13). This expression pattern may be partly related to developmental asynchrony among larvae after dsRNA treatment and the fact that the late sixth-instar larval stage represents a critical developmental window for the function of this target gene. Previous spatiotemporal qRT-PCR data indicated elevated expression of this gene from the sixth instar to the late sixth-instar larval stage, which is consistent with the qRT-PCR results. At 48 h, the silencing efficiency reached 71.4% (expression reduced to 28.6% of the control) and remained at 58% at 72 h. Nevertheless, no developmental abnormalities were observed in the larvae during this period. During the late sixth-instar larval stage, silencing efficiency peaked at 73.4%, accompanied by developmental anomalies. These findings suggest that feeding ds*SfCDA6* may achieve RNA interference of the target gene.

#### 3.6.4. Phenotypic Changes in *S. frugiperda* Following Feeding of *SfCDA6* dsRNA

Following ingestion of CNPs-ds*SfCDA6*-treated maize leaves, larval development of *S. frugiperda* proceeded without visible defects; phenotypic alterations were restricted to the ate sixth-instar larval and pupal phases. Upon entering the late sixth-instar larval period, treated insects exhibited pronounced body contraction, cuticular sclerotization, and darkened brownish-black pigmentation compared with controls. Specifically, the thoracic legs turned dark brown, while the abdominal prolegs also became brown and desiccated, accompanied by a pronounced decline in overall mobility (Figure 14A,B). Observations at the pupal stage revealed that control pupae exhibited normal morphology, whereas the treated group showed significant molting failure during pupation, characterized by incomplete shedding of the old cuticle from the head and legs, with portions of the exuvium adhering to the pupal body (Figure 14C,D). This ecdysis disruption ultimately led to pupal mortality of 53% (Figure 15).

## 4. Discussion

Given that CDA serves as a pivotal enzyme in the chitin modification pathway, contributing not only to the routine maintenance of the cuticle and intestinal tract but also playing essential roles during critical metamorphic events, members of this gene family are candidate genes for exploring RNAi-based pest control. In the present study, seven *CDA* genes were identified from the genome of *S. frugiperda*. Generally, the number of *CDA* genes in insects is limited. For instance, Dixit reported nine in *Tribolium castaneum* [44], Wu described nine in *Spodoptera exigua* [45], Xi identified four in *Nilaparvata lugens* [19], and Wang found two in *Panonychus citri* [46], indicating that the quantity of *CDA* genes varies among insect species.

Quantitative real-time PCR analysis revealed that *SfCDA2*, *SfCDA3*, and *SfCDA4* exhibited peak expression during the third larval instar, whereas *SfCDA6* maintained relatively high transcript levels throughout the larval, late sixth-instar larval, and pupal stages, consistent with findings in *Mamestra brassicae* [47]. The elevated expression of *SfCDA1* and *SfCDA7* in the fifth instar resembled the expression patterns of *SeCDA7* and *SeCDA9* in *S. exigua*. *SfCDA5* showed high expression during the pupal stage. Previous studies have demonstrated that *CDA* genes display distinct tissue-specific expression patterns across different insects. In this work, *SfCDA1*, *SfCDA2*, *SfCDA3*, *SfCDA4*, *SfCDA5*, *SfCDA6*, and *SfCDA7* showed expression patterns in the cuticle consistent with those of *TcCDA1* and *TcCDA2* in *T. castaneum* [8], as well as *HvCDA1* and *HvCDA2* in *Heortia vitessoides* [48]. In *Mythimna separata*, CDA transcripts were broadly distributed across larval tissues, with the highest abundance in the midgut [49], a finding that aligns with the strong midgut expression of *SfCDA6* observed in this study. By contrast, in *H. armigera*, a species closely related to *S. frugiperda*, CDA expression was mainly detected in the fat body during the larval stage [34].

In recent years, RNAi technology has been explored as a potential strategy for insect pest management. In this study, chitosan-encapsulated dsRNA was delivered by injection to systematically evaluate the RNAi efficiency of seven *CDA* genes in *S frugiperda*. The results revealed that *SfCDA1*, *SfCDA4*, *SfCDA5* and *SfCDA7* were significantly downregulated at 24–72 h post-interference, while no obvious developmental malformations or growth abnormalities were observed in the treated larvae. The absence of prominent phenotypic defects following effective gene silencing is a common phenomenon in insect RNAi research, which can be attributed to multiple factors, including functional redundancy among genes, long protein half-life, incomplete gene silencing in target tissues, and other factors [50]. Several previous studies have reported similar observations: insects may not exhibit obvious phenotypic abnormalities even when target gene transcription is markedly suppressed. For example, Zhang et al. injected ds*BmCDA1* and ds*BmCDA2* into silkworm larvae and found significant reduction in target gene expression without obvious developmental defects [35]. Similarly, RNAi of *SfCDA3* in *Sogatella furcifera* caused strong suppression of gene expression but failed to induce visible phenotypic abnormalities [51]. In addition, Courtney Davis-Voge et al. reported that successful knockdown of *Ago2*, *Dcr1*, *Dcr2* and *Drosha* in western corn rootworm did not lead to significant fitness impairment or abnormal phenotypes; only silencing of a small subset of genes triggered obvious physiological defects [52]. These findings further confirm that transcriptional downregulation is not always coupled with phenotypic defects in insects.

In contrast, the interference effects on *SfCDA6* were more pronounced, with reduced transcript levels accompanied by evident phenotypic changes. Similarly, in *N. lugens* [19], *CDA1*, *CDA2*, and *CDA4* play critical roles, and injection of dsRNA targeting *NlCDA1*, *NlCDA2*, or *NlCDA4* resulted in cuticular abnormalities and elevated mortality. In *Laodelphax striatellus* [53], injection of dsRNA targeting *LsCDA1* and *LsCDA2* caused over 95% larval mortality and 100% egg mortality. Among the *SfCDA* genes, *SfCDA6* exhibited the most robust interference effects, with silencing efficiencies exceeding 80% at 72 h post-injection and mortality rates of 60%, respectively. Likewise, in *Heortia vitessoides*, injection of dsRNA targeting *HvCDA1* and *HvCDA2* resulted in mortality rates of 33% and 53%, respectively [48]. Following knockdown of *SfCDA6*, larvae displayed growth deformities, developmental delay, molting failure, and pupation difficulties, ultimately leading to death. Notably, once injected larvae experienced molting difficulties and failed to complete ecdysis within 48 h, death invariably followed. A study on *Tuta absoluta* also found that injection of dsRNA targeting *TaCDA1* increased mortality during the larva–pupa and pupa–adult transitions, indicating that the RNAi effects of *CDA* genes are concentrated at the molting and metamorphic stages [54].

Although injection-based RNAi yields strong effects, this delivery method is not directly applicable for field pest control. Interference via natural insect feeding offers greater practical potential. For instance, Liu and colleagues first employed chitosan nanoparticles to encapsulate dsRNA targeting the *BmToll9-2* gene in *Bombyx mori* [55]; after larval ingestion, silencing of *BmToll9-2* resulted in reduced body size in both larvae and pupae. This study provided direct genetic evidence for the role of chitosan nanoparticle-mediated feeding RNAi in insect metamorphosis and body size regulation. In the present work, the *SfCDA6* gene, which exhibited both high interference efficiency and pronounced phenotypic effects, was selected for further chitosan-mediated feeding experiments. The results showed that feeding with ds*SfCDA6* led to a peak silencing efficiency of 73.4% at the late sixth-instar larval stage, yet no obvious developmental abnormalities were observed during the larval period. Growth defects and molting difficulties emerged only at the late sixth-instar larval stage, ultimately resulting in pupation failure and death. This stage-specific lethal effect may be attributed to the spatiotemporal expression pattern of *SfCDA6*: the gene is predominantly expressed from the sixth instar through the late sixth-instar larval stage, and its encoded protein participates in body wall formation and remodeling during pupation. Consequently, the silencing effect becomes evident precisely when the functional demand for this protein is highest. This finding is consistent with studies on lipase and chitinase genes in *H. armigera* [56], where ingestion of chitosan-encapsulated dsRNA nanoparticles severely impaired pupation and adult emergence, with zero successful eclosion. This observation suggests that when screening RNAi targets for pest control, focusing solely on larval phenotypic changes may overlook genes that play critical roles during late metamorphosis. Collectively, the qRT-PCR, injection, and feeding experiments further confirm the essential function of *CDA* genes in the insect molting process.

From the perspective of pest management, On the one hand, ds*SfCDA6* feeding interference can induce high pupal mortality of *S. frugiperda*, verifying that *SfCDA6* is a viable and effective RNAi target for pest suppression, on the other hand, the lethal effect of ds*SfCDA6* mainly occurs in the late larval and pupal stages, and insect feeding damage during the larval period cannot be effectively eliminated, which limits its independent application in field pest control. Accordingly, *SfCDA6* is more suitable for combined RNAi with key functional genes acting in the larval stage, such as chitin synthase and digestive system-related genes, to realize synergistic pest control throughout the whole developmental cycle of *S. frugiperda*.

Despite the stable and credible phenotypic results obtained from both injection and feeding RNAi assays in this study, several inherent limitations remain. First, although *SfCDA6* knockdown triggered prominent molting and pupation defects, only one dsRNA fragment was used for functional validation of *SfCDA6*, and a second non-overlapping dsRNA is required to further confirm the phenotypic results. The current study lacks biochemical and ultrastructural evidence, including quantitative detection of chitin content, determination of CDA enzymatic activity, and observation of cuticle ultrastructure. The absence of these data fails to fully elaborate the precise molecular mechanism by which *SfCDA6* participates in cuticle remodeling. Second, *SfCDA6* is highly expressed in both the epidermis and midgut, indicating its potential involvement in peritrophic matrix formation and digestive physiology. Thus, the observed lethal phenotypes may originate from both epidermal structural damage and impaired midgut physiological functions, rather than simply abnormal cuticle development. To address the above limitations and further support the optimization of RNAi target combinations based on the chitin metabolism pathway, future studies will focus on clarifying the specific regulatory mechanism of *SfCDA6* in the chitin modification pathway. Specifically, we will detect the enzymatic activity of *SfCDA6* and quantify chitin structural changes after gene silencing to confirm its exact function in chitin deacetylation and cuticle remodeling. Ultrastructural observation of epidermal and midgut tissues will also be performed to distinguish the dual biological functions of *SfCDA6* in cuticle formation and peritrophic matrix maintenance. Furthermore, exploring the functional compensation and interaction relationships among different SfCDA family members will help systematically elucidate the regulatory network of insect chitin metabolism and provide a solid theoretical foundation for developing efficient multi-gene synergistic pest control strategies.

## 5. Conclusions

In this study, a comprehensive analysis of *CDA* genes in *S. frugiperda* was performed. Seven *SfCDA* genes were identified, and phylogenetic analysis indicated close evolutionary relationships with *S. litura* and *H. armigera*. qRT-PCR showed that most *SfCDA* genes were highly expressed in the third and fifth instars and in the integument. Among them, *SfCDA6* showed the largest fold change relative to its own egg expression from the third to sixth instars, with peaks at the third and sixth instars, and high abundance in both midgut and cuticle. Injection-based RNAi revealed that silencing *SfCDA2*, *SfCDA3*, or *SfCDA6* caused molting abnormalities, with *SfCDA6* producing the most severe lethal phenotypes. Following feeding of ds*SfCDA6*, the strongest silencing was observed at the late sixth-instar larval stage, leading to body shrinkage, cuticular hardening, and pigmentation anomalies in late sixth-instar larvae, ultimately resulting in pupation failure and death. These findings confirm that *SfCDA6* plays a critical role in the late metamorphosis of *S. frugiperda*, providing a theoretical basis for screening RNAi targets within the chitin modification pathway.

## Figures and Tables

**Figure 1:**
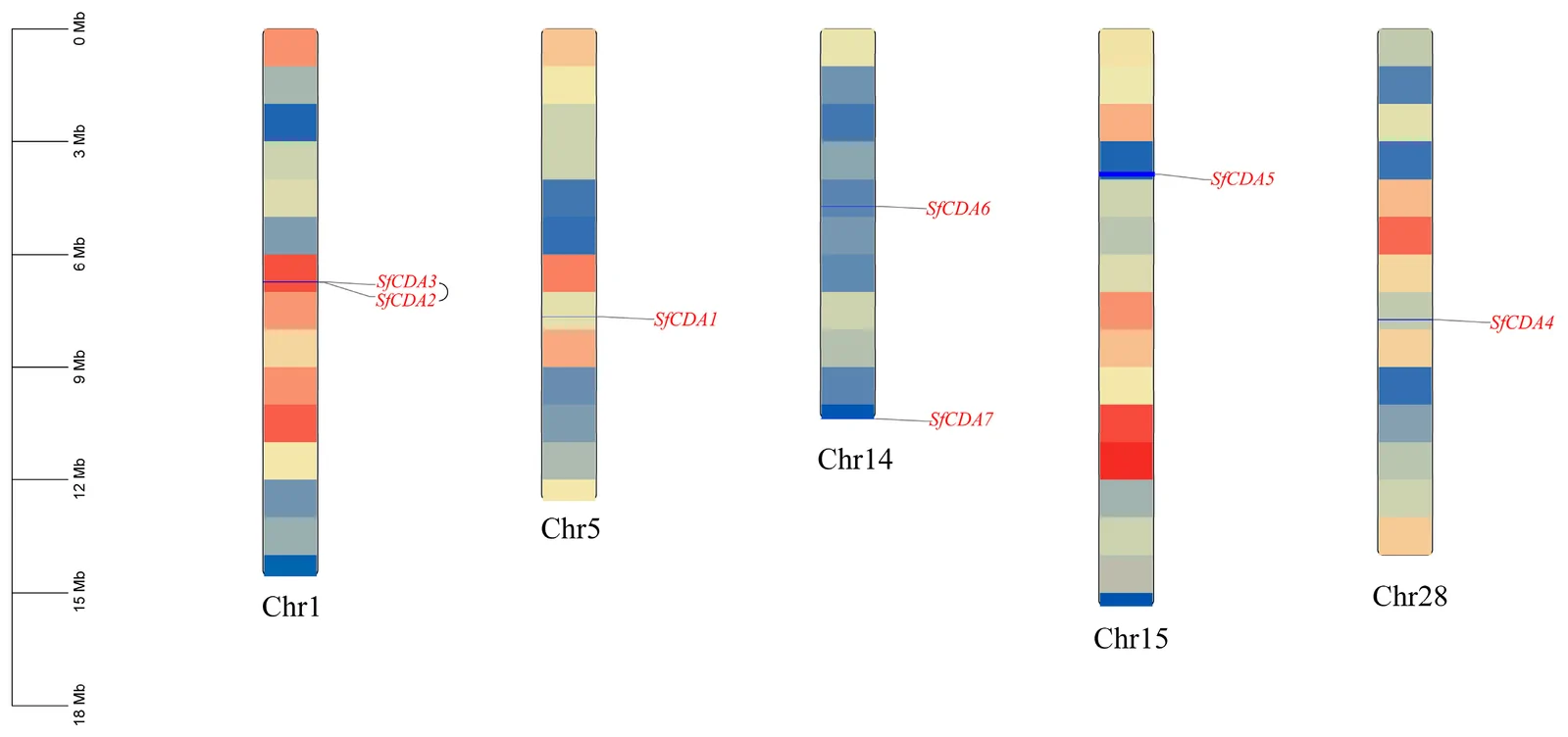
Chromosomal localization and gene duplication analysis of *SfCDA* genes. The color gradient from light to dark along the chromosomes corresponds to low to high gene density.

**Figure 2:**
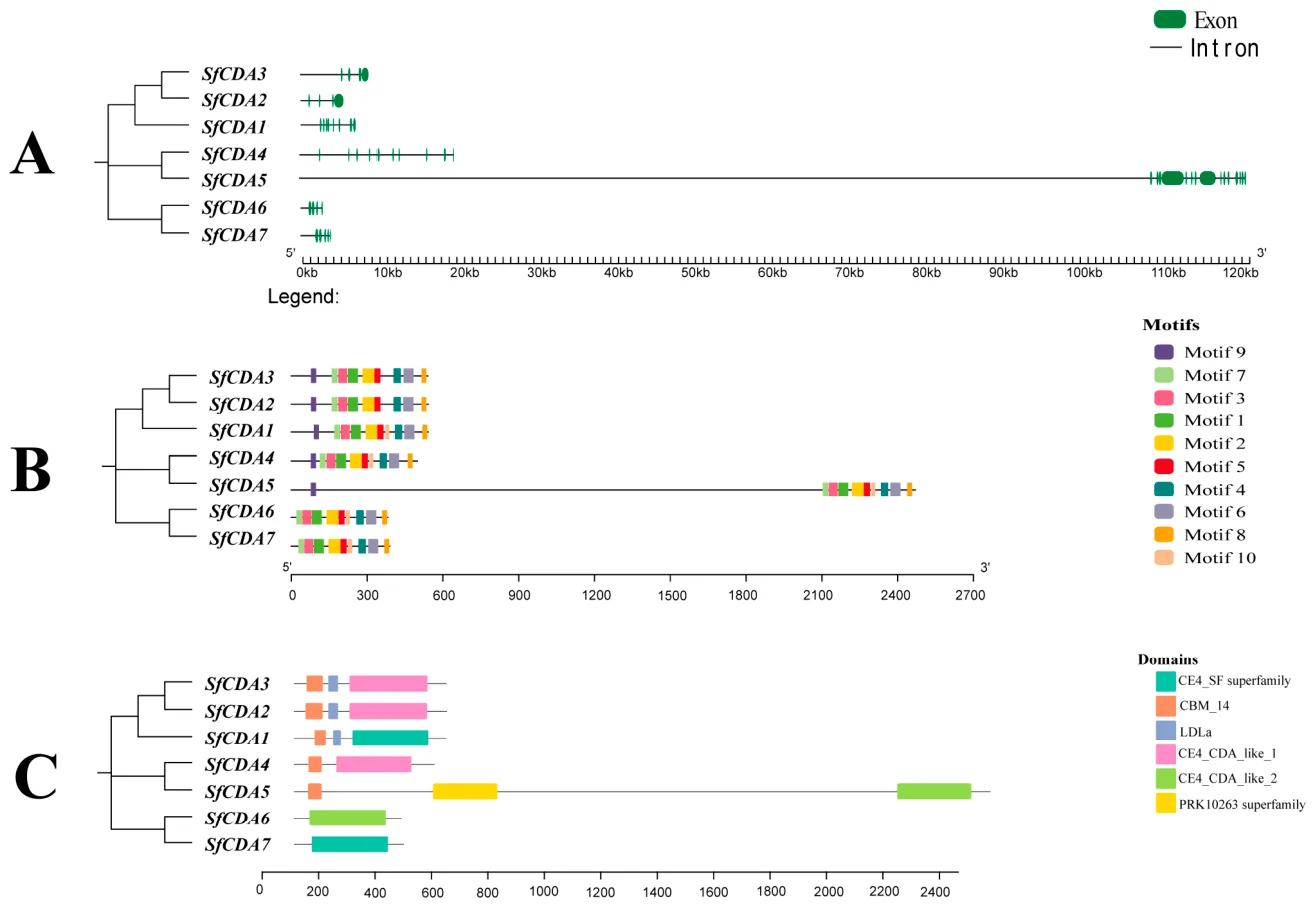
Structural and conserved motif analyses of *SfCDA* genes. (**A**) Exon–intron structure of *SfCDA* genes. (**B**) Conserved motif model of *SfCDA* genes. (**C**) Conserved domains of *SfCDA* genes.

**Figure 3:**
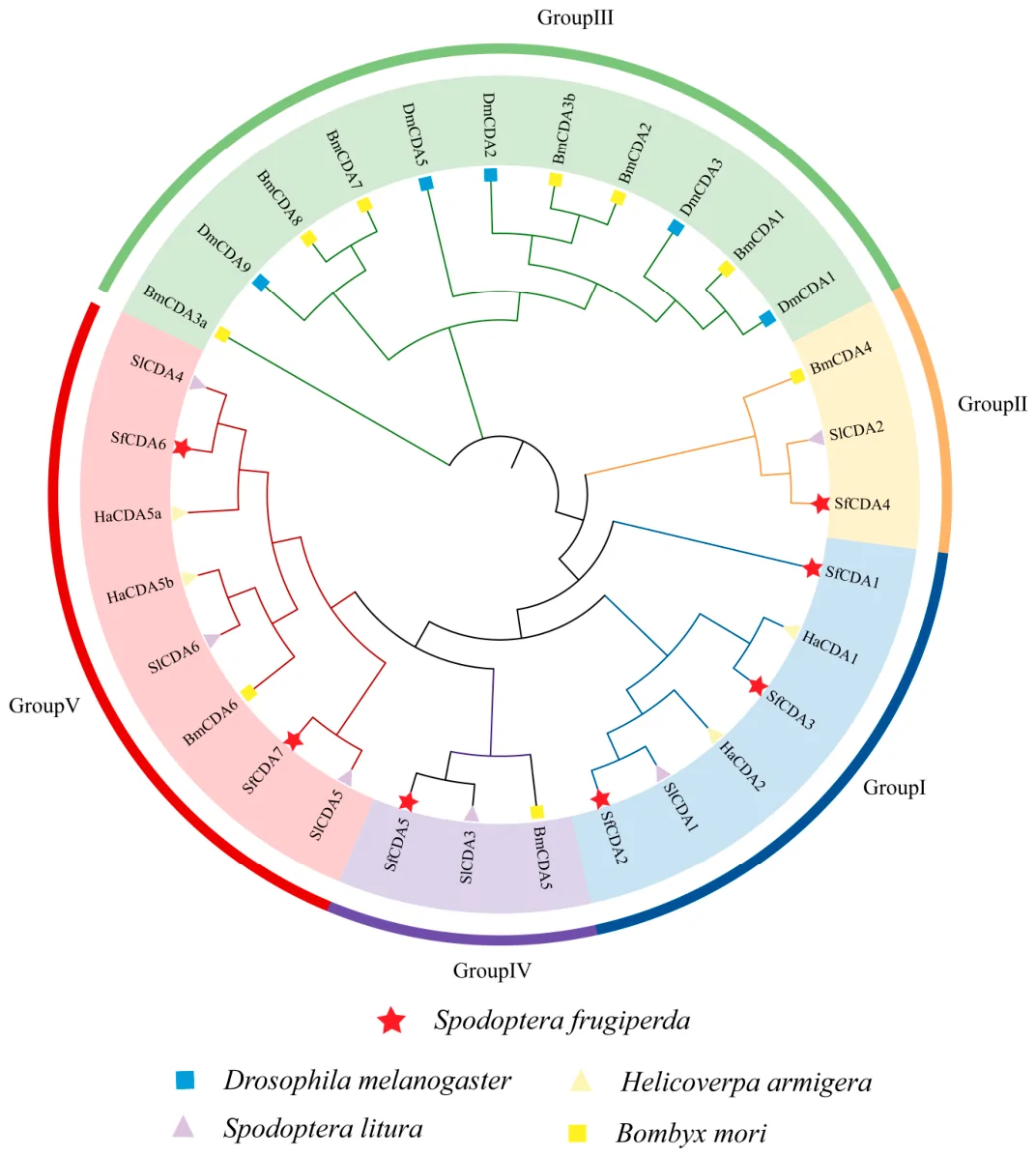
Phylogenetic tree of CDA proteins from *S. frugiperda*, *Spodoptera litura*, *Helicoverpa armigera*, *Bombyx mori*, and *Drosophila melanogaster*. The branch lengths represent genetic distances.

**Figure 4:**
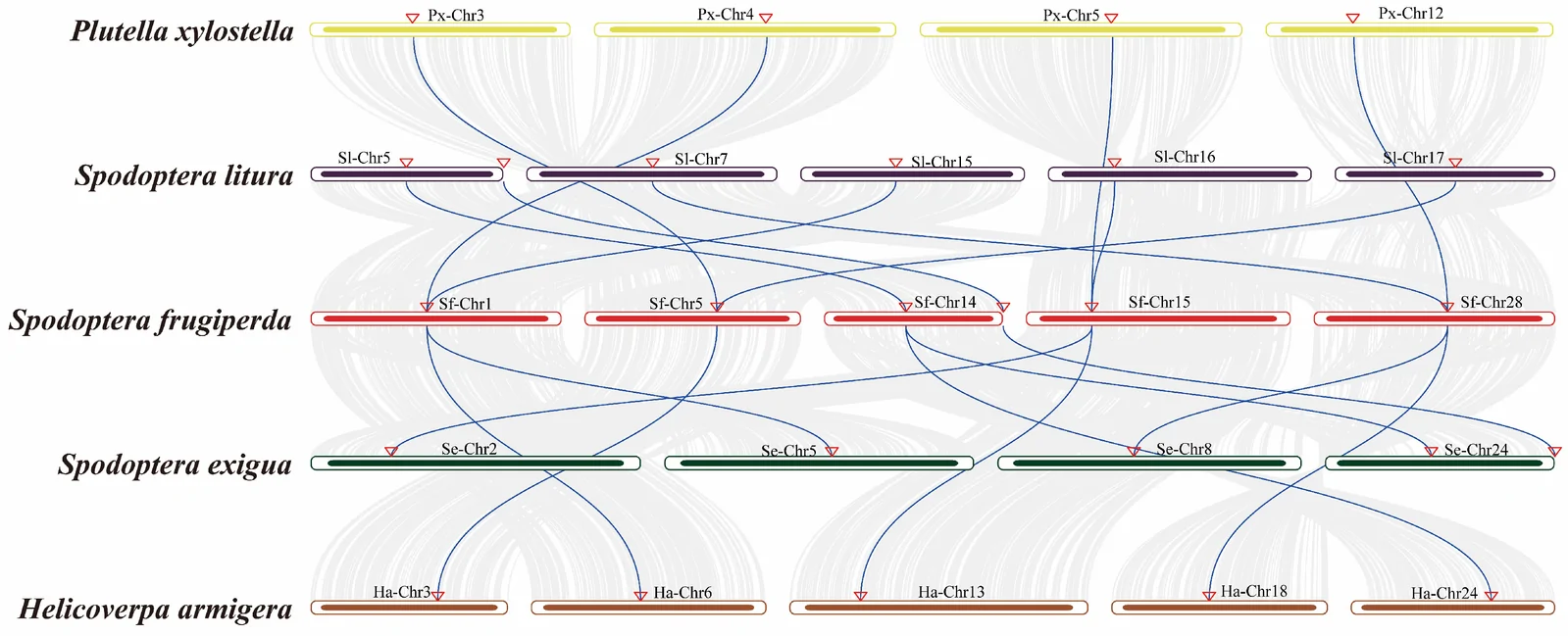
Synteny analysis of *SfCDA* genes. Lines indicate collinear relationships between species, and triangles mark gene positions.

**Figure 5:**
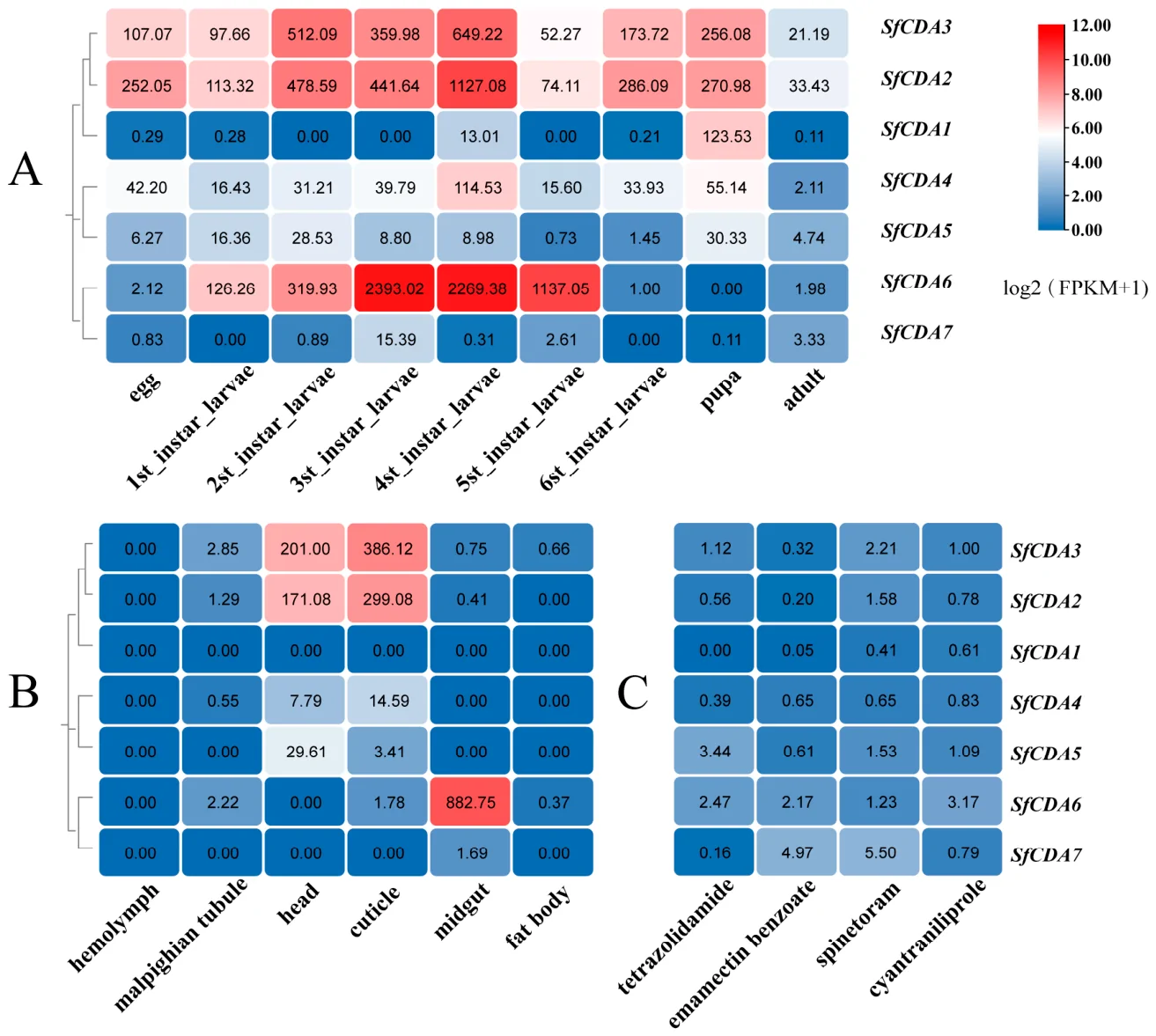
Transcriptomic analysis of *SfCDA* genes. Expression heatmaps of the *SfCDA* gene family in *S. frugiperda* across different developmental stages, tissues, and insecticide treatments. (**A**) Transcriptomic data analysis of *SfCDA* genes at different developmental stages of *S. frugiperda*. (**B**) Transcriptomic data analysis of *SfCDA* genes in different tissues of *S. frugiperda*. (**C**) Transcriptomic data analysis of *SfCDA* genes under different insecticide treatments. The color scale represents Log_2_ (FPKM+1) values. Higher Log_2_ (FPKM+1) values indicate greater transcript abundance.

**Figure 6:**
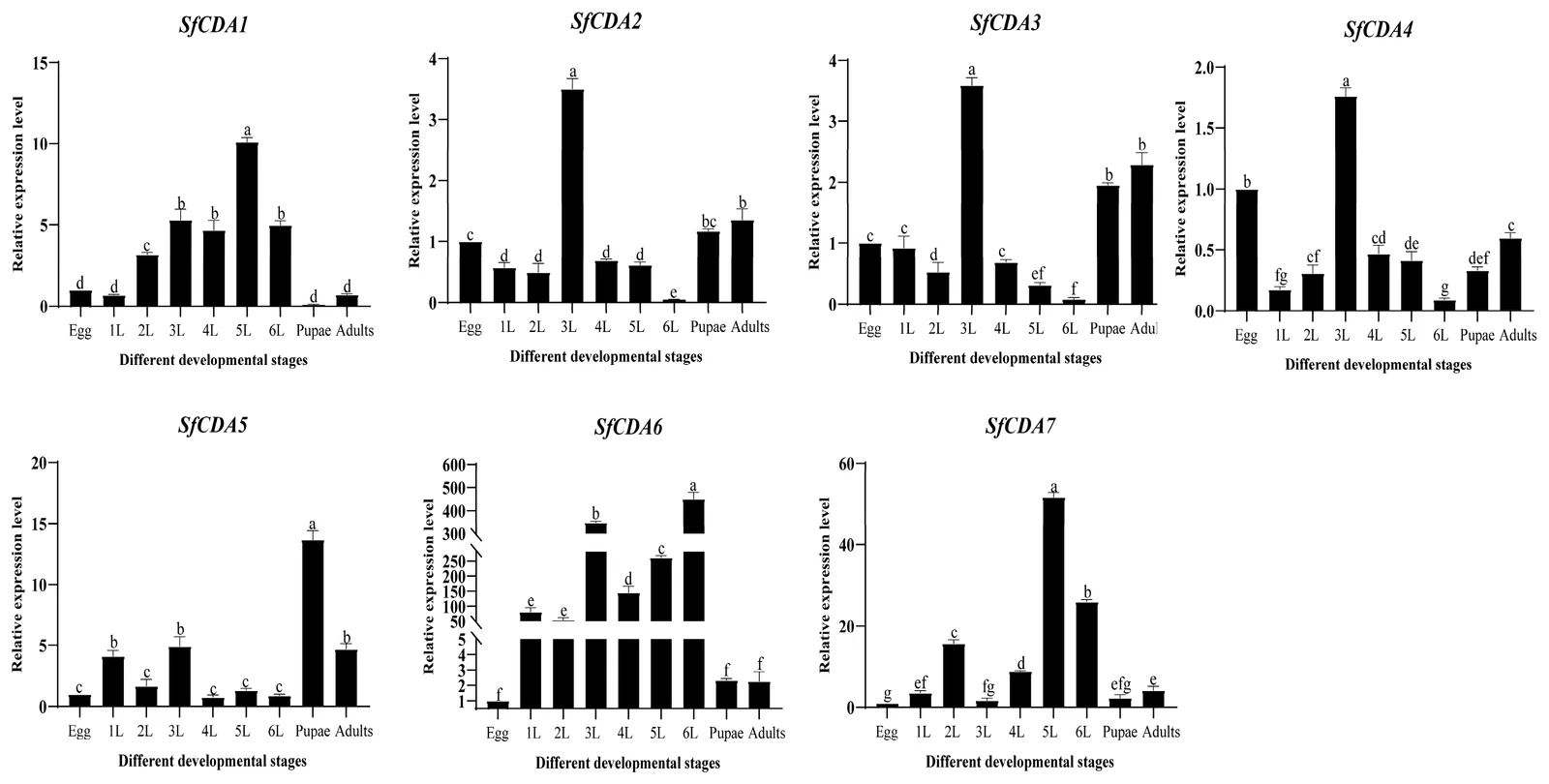
Expression of *SfCDA* genes at different developmental stages of *S. frugiperda*. egg:egg stage; 1L: 1st instar stage; 2L: 2nd instar stage; 3L: 3rd instar stage; 4L: 4th instar stage; 5L: 5th instar stage; 6L: 6th instar stage; Pupae: pupal stage; Adults: adult stage. Data are presented as mean ± standard deviation. Using the egg stage as a reference, the expression levels of the seven *SfCDA* genes at different developmental stages was evaluated. Different letters above the bar chart indicate statistically significant differences, (*p* < 0.05).

**Figure 7:**
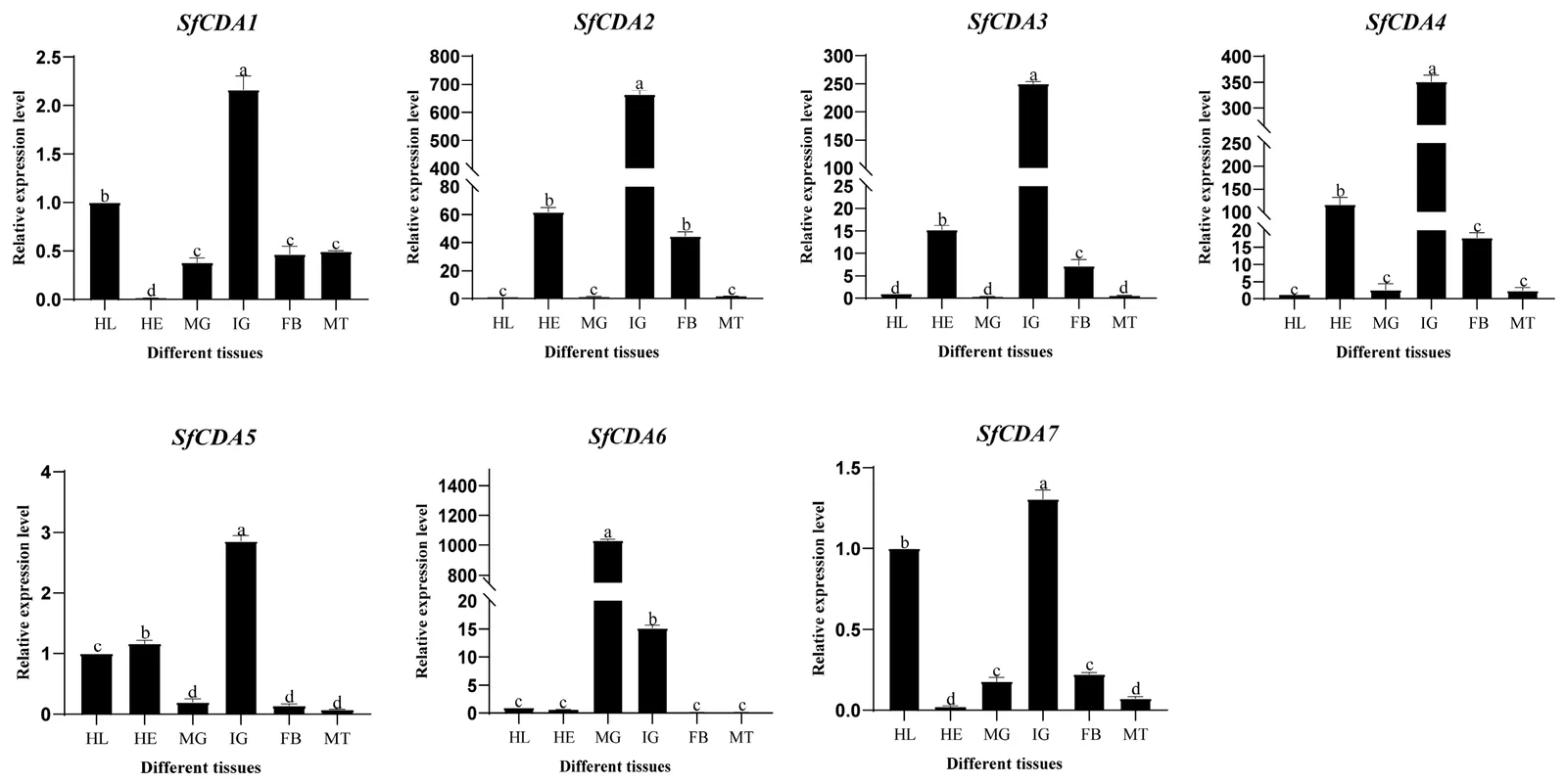
Expression of *SfCDA* genes in different tissues of *S. frugiperda*. MT: Malpighian tubules; MG: midgut; HE: head; IG: integument; FB: fat body; HL: hemolymph. Data are presented as mean ± standard deviation. Different letters above the bars indicate statistically significant differences (*p* < 0.05).

**Figure 8:**
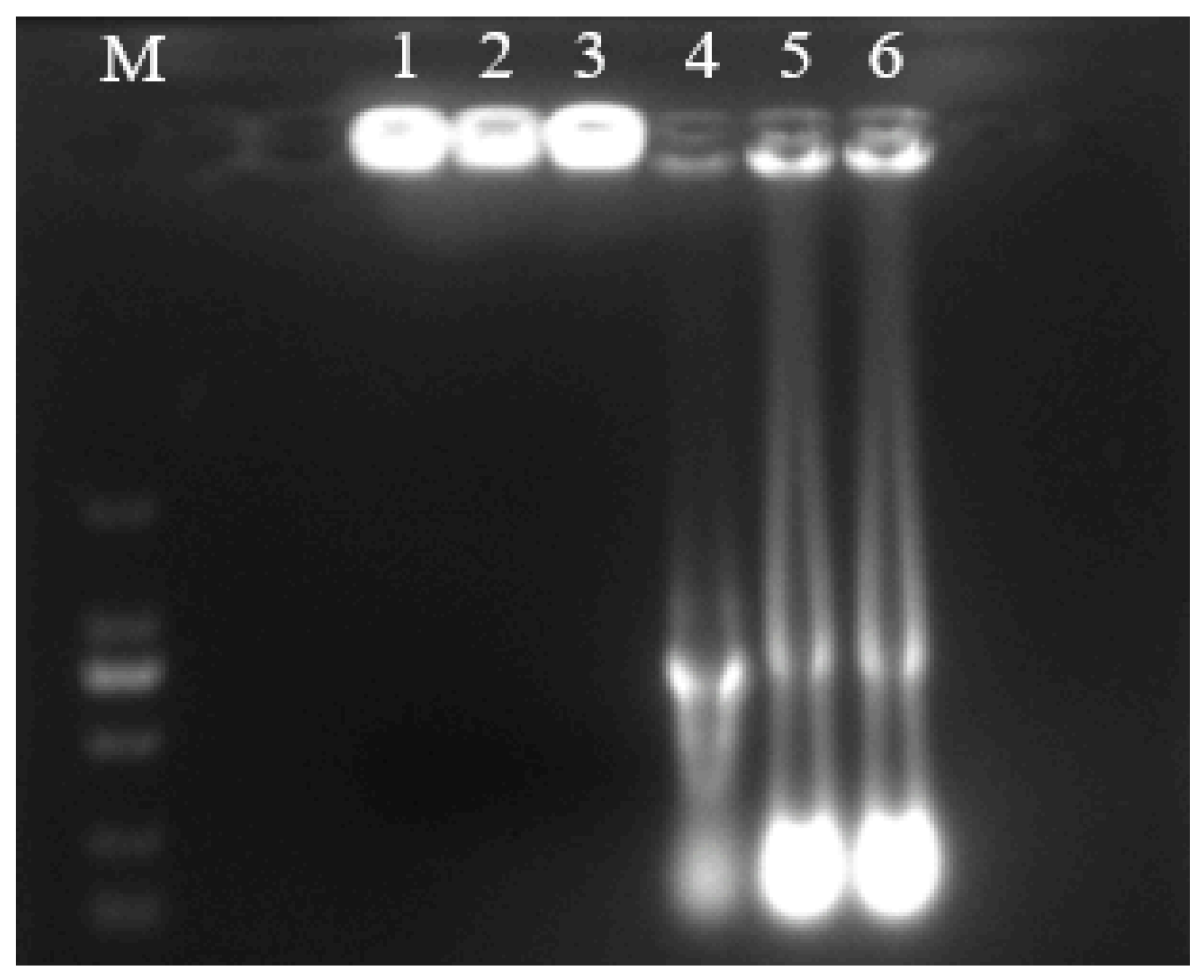
Agarose gel retardation assay confirming the formation of CNP-dsRNA complexes. Lanes 1–3: CNP-dsRNA complex; Lanes 4–6: naked dsRNA control.

**Figure 9:**
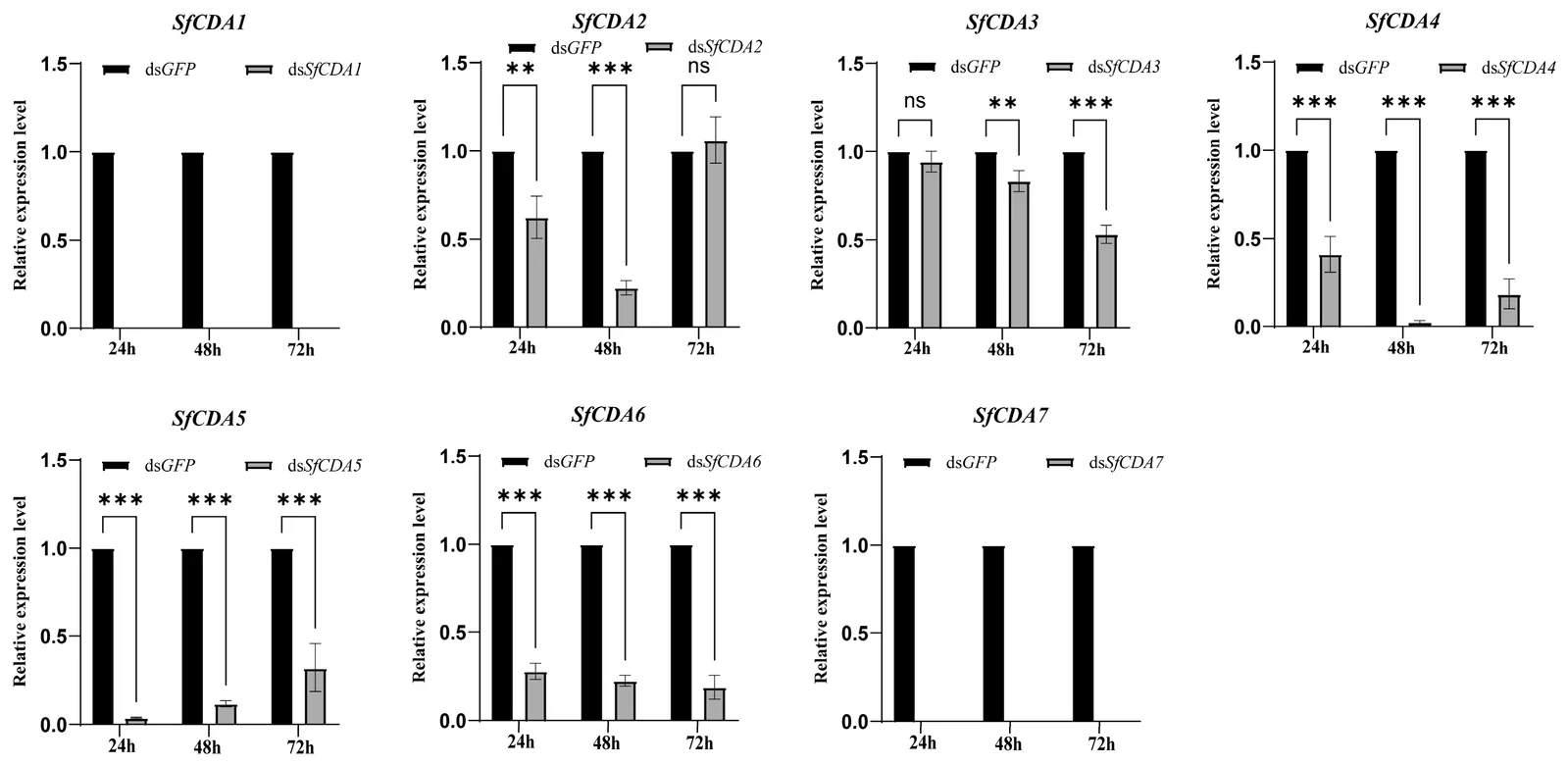
Silencing efficiency of RNAi targeting *SfCDA* genes. Uniform day-1 fifth-instar larvae (within 12 h after molting) were used for injection of all seven *SfCDA* genes. Data were analyzed using two-way ANOVA to evaluate the effects of treatment, time and their interaction, followed by Sidak’s multiple comparison test. Asterisks indicate significant differences in gene expression levels compared with the ds*GFP* control group at the corresponding time point. ** *p* < 0.01, *** *p* < 0.001; ns indicates no significant difference (*p* > 0.05). Data are presented as mean ± SEM (*n* = 3 biological replicates).

**Figure 10:**
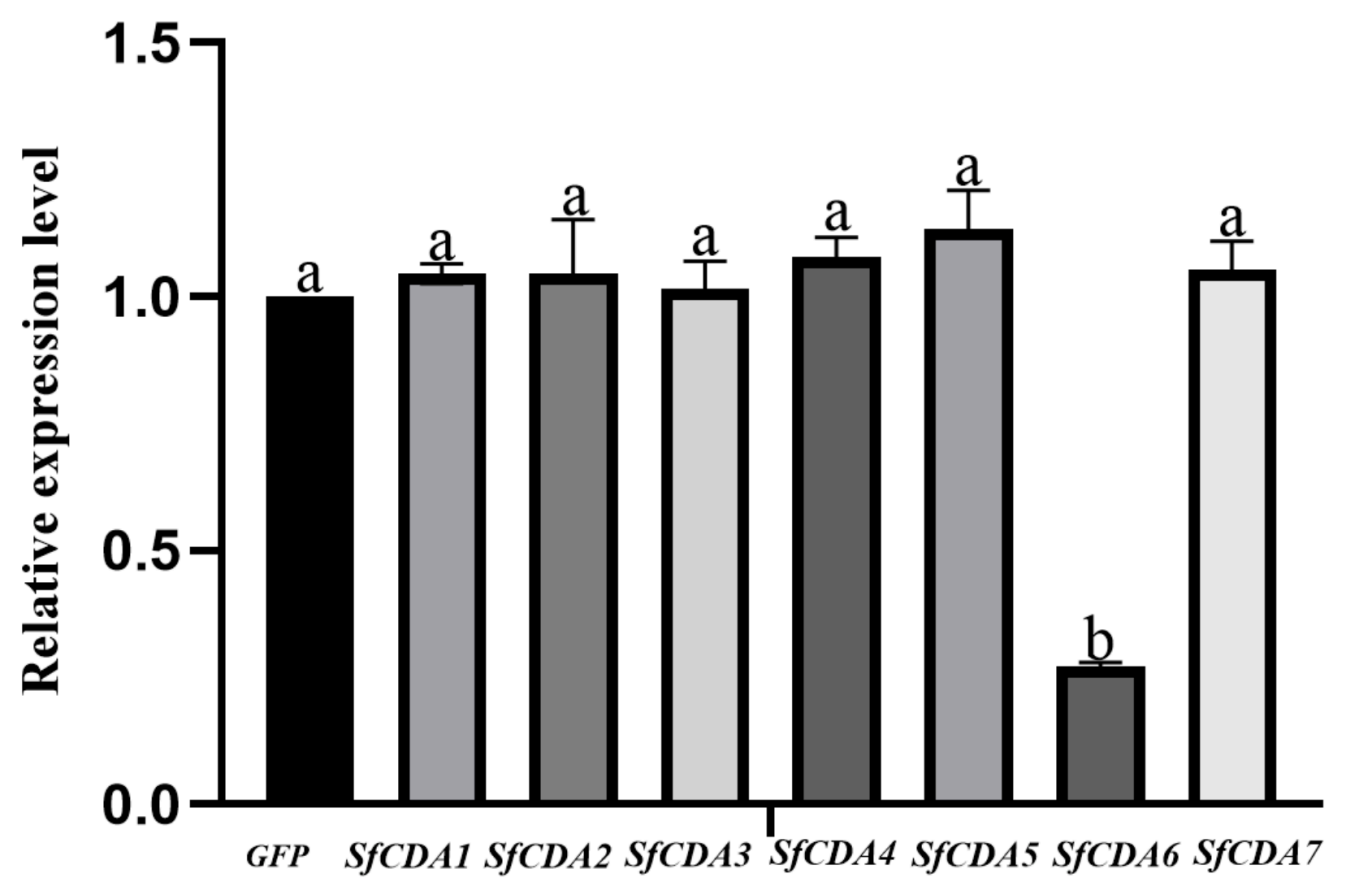
Relative expression levels of *SfCDA* family genes in larvae at 72 h post ds*SfCDA6* injection. Data were analyzed by one-way ANOVA followed by Tukey’s HSD multiple comparison test. Different lowercase letters above bars indicate statistically significant differences (*p* < 0.05). Error bars represent the standard error of the mean (SEM, *n* = 3 biological replicates).

**Figure 11:**
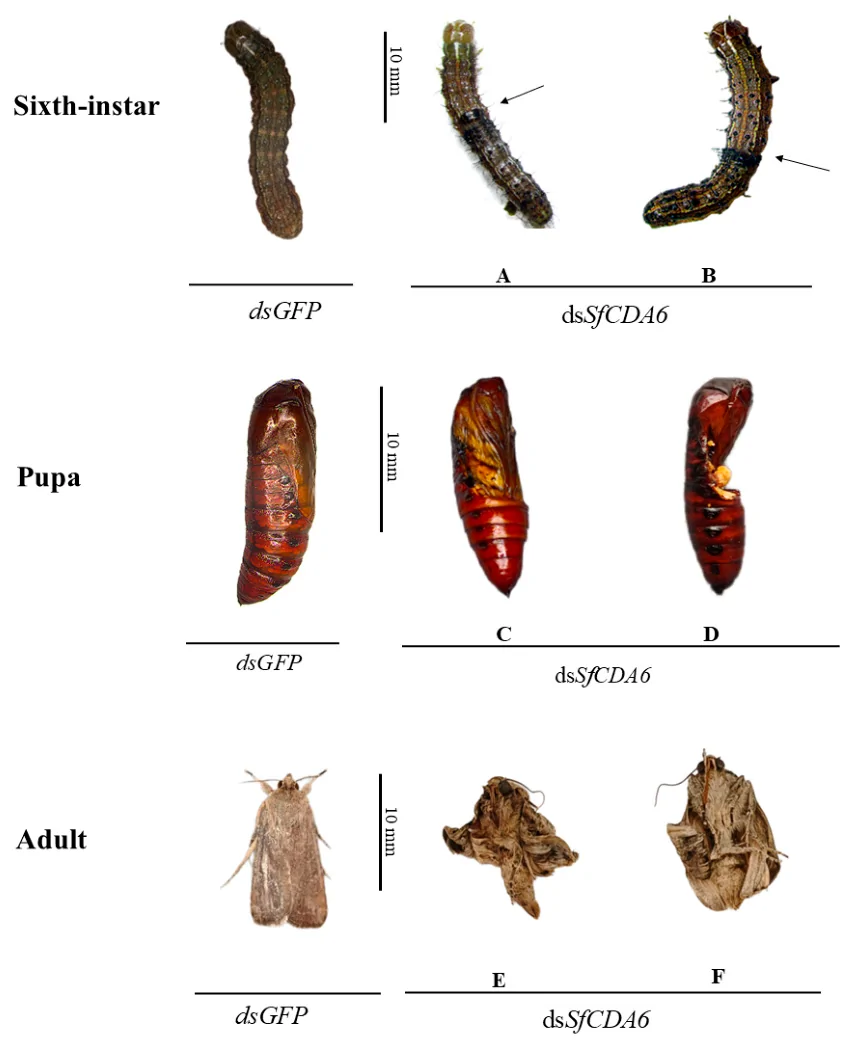
Phenotypic changes in *Spodoptera frugiperda* fifth-instar larvae after injection with CNPs-ds*SfCDA6*. Control: larvae injected with CNPs-ds*GFP*; ds*SfCDA6* group: larvae injected with CNPs-ds*SfCDA6*. (**A**,**B**) Abnormal development at the sixth-instar larval stage; (**C**,**D**) abnormal development at the pupal stage; (**E**,**F**) abnormal development at the adult stage. Phenotypic frequencies: molting defects observed at 2 days post-injection (16.6%), with the arrow indicating the site of molting failure; pupal death at 5 days post-injection (26.7%); adult death at 9 days post-injection (16.7%), total mortality 60.0% (*n* = 30). Scale bar = 10 mm.

**Figure 12:**
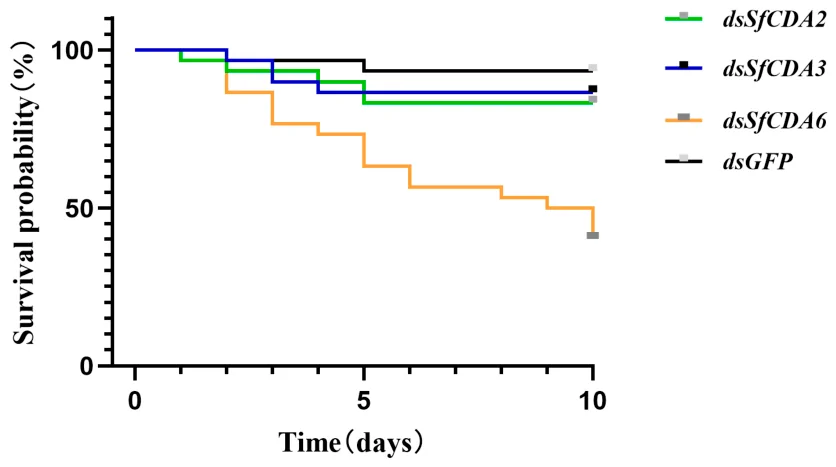
Kaplan–Meier survival curves showing the survival probability of *S. frugiperda* larvae over 10 days after injection with CNPs-ds*GFP*, CNPs-ds*SfCDA2*, CNPs-ds*SfCDA3* and CNPs-ds*SfCDA6*. Larvae injected with CNPs-ds*GFP* served as the control group. The horizontal axis represents time (days) post-injection, and the vertical axis represents estimated survival probability (%). The final mortality rates were 6.6% for CNPs-ds*GFP*, 13.3% for CNPs-ds*SfCDA3*, 16.6% for CNPs-ds*SfCDA2*, and 60% for CNPs-ds*SfCDA6*. Survival differences among groups were compared using the log-rank test. Each experiment was repeated three times with 10 individuals per replicate. Data are presented as mean ± SEM from three independent experiments.

**Figure 13:**
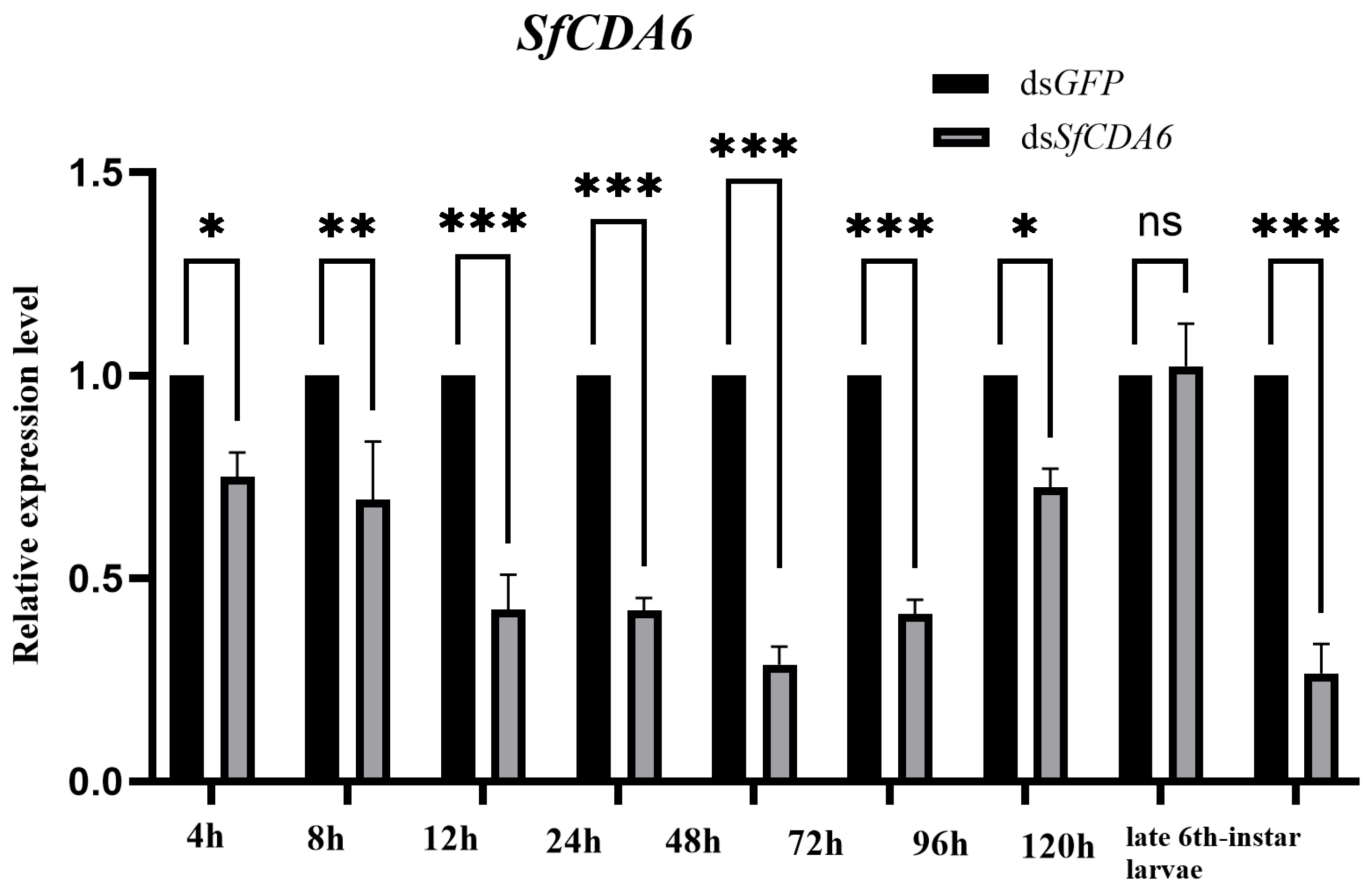
Silencing efficiency of RNAi targeting the *SfCDA6* gene. *SfCDA6* Data were analyzed using two-way ANOVA to evaluate the effects of treatment, time and their interaction, followed by Sidak’s multiple comparison test. Asterisks indicate significant differences in gene expression levels compared with the ds*GFP* control group at the corresponding time point * *p* < 0.05, ** *p* < 0.01, *** *p* < 0.001; ns indicates no significant difference (*p* > 0.05). Data are presented as mean ± SEM (*n* = 3 biological replicates).

**Figure 14:**
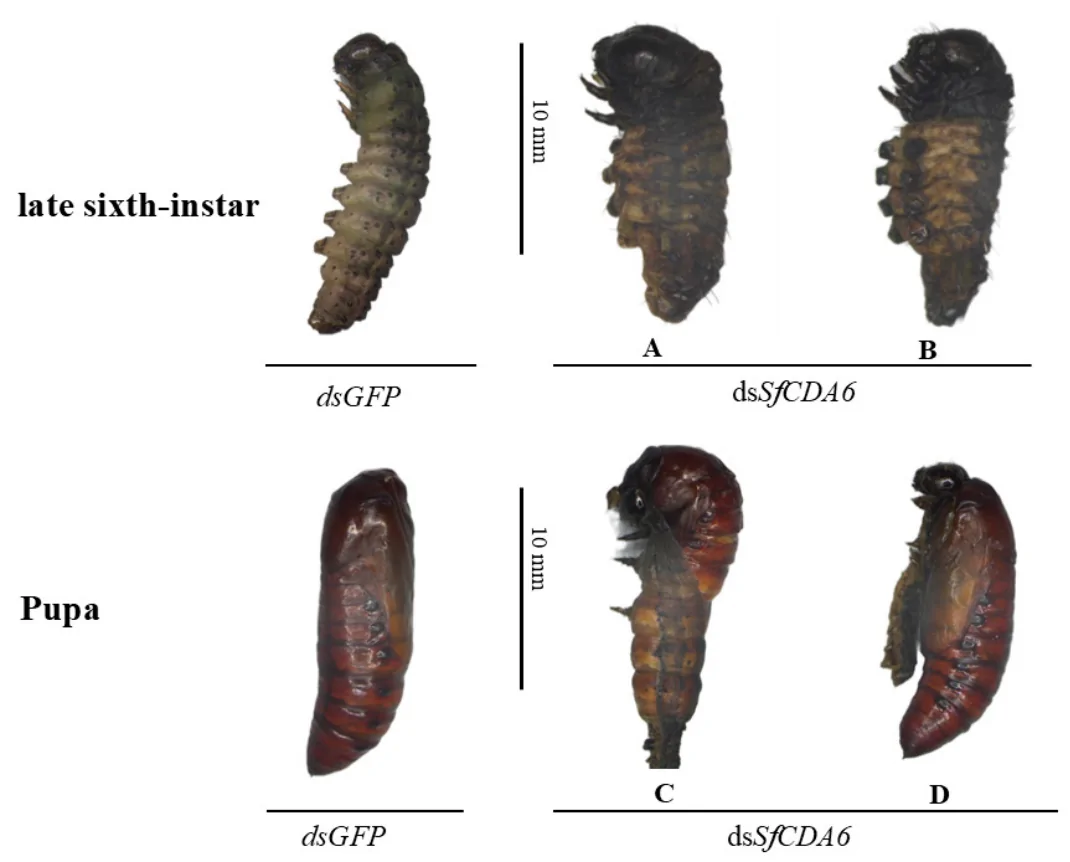
*SfCDA6* Phenotypic changes in *S. frugiperda* larvae after feeding treatment. Control: larvae fed with CNPs-ds*GFP*; ds*SfCDA6* group: larvae fed with CNPs-ds*SfCDA6*. (**A**,**B**) Abnormal development in late sixth-instar larvae; (**C**,**D**) Abnormal development at the pupal stage. Phenotypic frequencies: abnormal pupation in late sixth-instar larvae observed at 9 days post-feeding (40%), pupal death at 11 days post-feeding (13%), total mortality 53% (*n* = 30). Scale bar = 10 mm.

**Figure 15:**
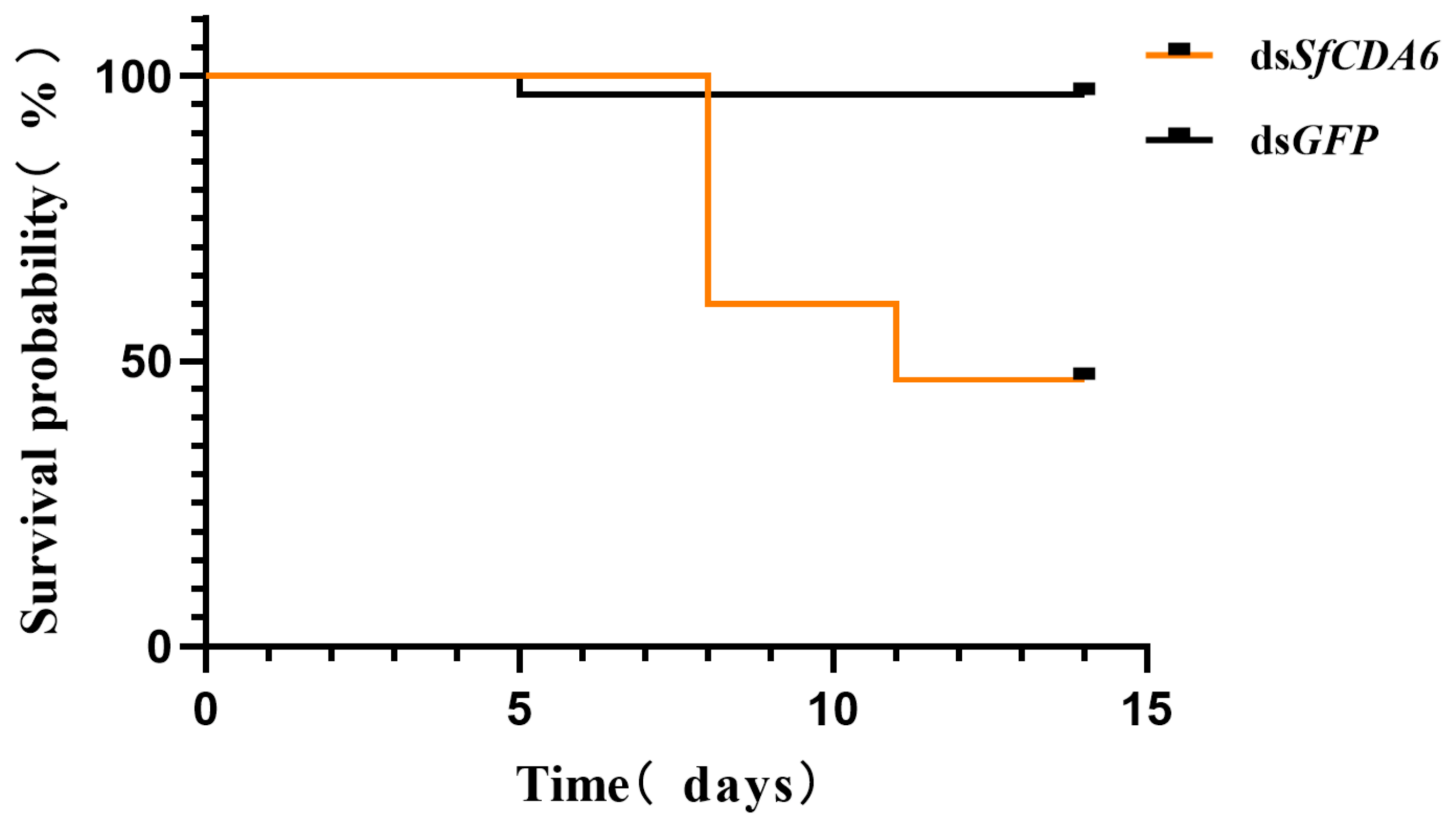
Kaplan–Meier survival curves showing the survival probability of *Spodoptera frugiperda* larvae over 15 days after feeding with CNPs-ds*GFP* and CNPs-ds*SfCDA6*. Larvae fed with CNPs-ds*GFP* served as the control group. The horizontal axis represents time (days) post-feeding, and the vertical axis represents estimated survival probability (%). The final mortality rate was 53.0% for the CNPs-ds*SfCDA6* group. Survival differences between groups were compared using the log-rank test. Each experiment was repeated three times with 10 individuals per replicate. Data are presented as mean ± SEM from three independent experiments.

**Table 1:** List of primers used for qRT-PCR in this study.

Primer Name	Primer Sequences (5′-3′)	Length (bp)
*RPL3*	F: AAGCCAGTCCACCTTACCGC	206
	R: GCCCAAACAGTGAGCAGAGC	
*SfCDA1*	F: CGCCACTGTCTGATCCTCCATT	104
	R: TCCTGCGTAGCTTCGTGTTGG	
*SfCDA2*	F: GCGTGTCTGTGTCTTGGTGTGT	89
	R: TCATCTCCTCCGTCCTCCTCCT	
*SfCDA3*	F: GGGCAAAGAAATGGCGGGTATG	94
	R: ACGAAGGTAAGGAGCACGAACA	
*SfCDA4*	F: GGCTGTCCCATTAGAGGCACTT	122
	R: AGACCTTGCTGCTGCGATACC	
*SfCDA5*	F: ACTACCCACATCCCACCGACTG	141
	R: TGCCGTCACAGCCTACATTGC	
*SfCDA6*	F: CTTGTGTCCGCTGTGGCACTT	109
	R: CAGGAATGGCTTGGCTGGAACA	
*SfCDA7*	F: GTCGCACTTCGCCAACATTCC	97
	R: GTCCGCCATCACCCTGTAACTG	

**Table 2:** List of primers used for RNAi in this study.

Primer Name	Sequence of Primers (5′-3′)	Target Region	Length (bp)
ds*GFP*	TAATACGACTCACTATAGGGAAGTTCAGCGTGTCCG	/	/
	TAATACGACTCACTATAGGGCACCTTGATGCCGTTC		
ds*SfCDA1*	TAATACGACTCACTATAGGGGGCTGGTGCGATGTGAATTACG	490–699	210
	TAATACGACTCACTATAGGGCGGAGTGAACAGATGCTTCGTG		
ds*SfCDA2*	TAATACGACTCACTATAGGGCTGGTGGTCTCGCCTTCGATAT	242–458	217
	TAATACGACTCACTATAGGGTCGTCAGACTCATCCTTGCAGT		
ds*SfCDA3*	TAATACGACTCACTATAGGGAGCTATGCAAGGACAAGGACGC	128–396	269
	TAATACGACTCACTATAGGGGGAGTCACCACAAGCCAGGAAT		
ds*SfCDA4*	TAATACGACTCACTATAGGGTGAAGGAACACAGGGCAATGGA	144–379	236
	TAATACGACTCACTATAGGGGGCATTCCGCAGGATCACACT		
ds*SfCDA5*	TAATACGACTCACTATAGGGCTCCTGGAAAGAATTGGGCCTT	1076–1295	220
	TAATACGACTCACTATAGGGTGCGATTGAGAATTGCCTGACT		
ds*SfCDA6*	TAATACGACTCACTATAGGGCGGCGATGTCTTCGAGTTCCAT	249–498	250
	TAATACGACTCACTATAGGGGCACCAGCCTTCGTCAGATGAA		
ds*SfCDA7*	TAATACGACTCACTATAGGGAGTTCGTGGTGGTGGCGTTC	191–397	207
	TAATACGACTCACTATAGGGGGTTCATGTGCGAGATGGAGTG		

**Table 3:** Analysis of physicochemical properties of the *SfCDA* gene family.

Sequence ID	Gene IDs	RefSeq mRNA	Number of Amino Acid	Molecular Weight	Theoretical pI	Instability Index	Aliphatic Index	Grand Average of Hydropathicity
*SfCDA1*	LOC118272095	XM_035588427.2	541	62,721.42	5.96	47.01	66.32	−0.487
*SfCDA2*	LOC118273685	XM_035590780.2	542	61,522.51	5.31	47.79	69.78	−0.505
*SfCDA3*	LOC118273193	XM_035590027.2	539	61,433.97	5.03	47.49	66.60	−0.544
*SfCDA4*	LOC118282125	XM_050705934.1	498	56,260.77	5.40	36.74	66.22	−0.432
*SfCDA5*	LOC118279275	XM_050698993.1	2469	278,460.2	7.67	57.78	55.42	−0.956
*SfCDA6*	LOC118279197	XM_035598759.2	381	43,014.79	5.00	36.84	75.72	−0.129
*SfCDA7*	LOC126911362	XM_050698243.1	389	43,795.52	5.35	46.83	74.01	−0.213

## Data Availability

The data presented in this study are available on request from the corresponding author.
